# The practice of intensive care in Latin America: a survey of academic intensivists

**DOI:** 10.1186/s13054-018-1956-6

**Published:** 2018-02-21

**Authors:** Ricardo Castro, Nicolas Nin, Fernando Ríos, Leyla Alegría, Elisa Estenssoro, Gastón Murias, Gilberto Friedman, Manuel Jibaja, Gustavo Ospina-Tascon, Javier Hurtado, María del Carmen Marín, Flavia R. Machado, Alexandre Biasi Cavalcanti, Arnaldo Dubin, Luciano Azevedo, Maurizio Cecconi, Jan Bakker, Glenn Hernandez, On behalf of the Latin-American Intensive Care Network - LIVEN (www.redliven.org)

**Affiliations:** 1 Departamento de Medicina Intensiva, Facultad de Medicina, Pontificia Universidad Catolica de Chile, Diagonal Paraguay #362, Santiago Centro, RM 8330077 Chile; 2 Hospital Español, Avenida General Garibaldi, 1729 esq., Rocha, Montevideo Uruguay; 3 Agencia Nacional de Investigación e Innovación (ANII), Montevideo, Uruguay; 4 Servicio de Terapia Intensiva. Hospital Alejandro Posadas, Avenida Presidente Arturo U. Illia, El Palomar, Buenos Aires Argentina; 5 Servicio de Terapia Intensiva, Hospital Interzonal de Agudos General San Martin de La Plata, Avenida 1 1794, Casco Urbano, La Plata, Buenos Aires B1904CFU Argentina; 6 Clinica Bazterrica and Clinica Santa Isabel, Billinghurst 2072 (esquina Juncal), Ciudad Autónoma de Buenos Aires, Argentina; 7 Departamento de Medicina Interna - Faculdade de Medicina, Universidade Federal do Rio Grande do Sul, Hospital de Clínicas de Porto Alegre, Rua Ramiro Barcelos, 2350 - Santa Cecilia, Porto Alegre, RS 90035-903 Brasil; 8 Escuela de Medicina, Universidad Internacional del Ecuador, Unidad de Cuidados Intensivos, Hospital Eugenio Espejo, Avenida Gran Colombia, Quito, 170136 Ecuador; 9 Department of Intensive Care Medicine, Fundación Valle del Lili - Universidad ICESI, Cali, Carrera 98 No. 18-49, Cali, Valle del Cauca Colombia; 10 Unidad de Cuidados Intensivos, Hospital Regional 1 Octubre, ISSSTE, Avenida Instituto Politécnico Nacional 1669. Colonia Lindavista, c.p., Delegación Gustavo A. Madero, Ciudad de México, 07300 México; 11 Anesthesiology, Pain and Intensive Care Department, Federal University of Sao Paulo, Rua Sena Madureira, 1500 - Clementino, São Paulo, SP 04021-001 Brasil; 12 Research Institute HCor, Hospital do Coração, Rua. Desembargador Eliseu Guilherme, 147 - Paraíso, São Paulo, SP 04004-030 Brasil; 13 Catedra de Farmacología, Facultad de Ciencias Médicas, Universidad Nacional de La Plata, Buenos Aires, Argentina; 14 Servicio de Terapia Intensiva, Sanatorio Otamendi y Miroli, Azcuénaga 894, CABA, C1115AAB Argentina; 15 Anesthesiology, Pain and Intensive Care Department, Federal University of Sao Paulo, Sao Paulo, Brazil; 16 Emergency Medicine Department, University of Sao Paulo, Hospital Sirio-Libanes, Rua Dona Adma Jafet, 91 - Vista, Sao Paulo, SP 01308-050 Brasil; 17 St. George’s University Hospitals NHS Foundation Trust, London, SW17 0QT UK; 18 Division of Pulmonary, Allergy, and Critical Care Medicine, Columbia University Medical Center, 630 West 168th Street, New York, NY 10032 USA; 19 Unidad de Paciente Critico Adultos, Hospital Clinico UC-CHRISTUS, Marcoleta #367, Santiago Centro, RM 8330077 Chile

**Keywords:** Intensive care units, Latin American, LMIC, Critical care, Health, Manpower

## Abstract

**Background:**

Intensive care medicine is a relatively young discipline that has rapidly grown into a full-fledged medical subspecialty. Intensivists are responsible for managing an ever-increasing number of patients with complex, life-threatening diseases. Several factors may influence their performance, including age, training, experience, workload, and socioeconomic context. The aim of this study was to examine individual- and work-related aspects of the Latin American intensivist workforce, mainly with academic appointments, which might influence the quality of care provided. In consequence, we conducted a cross-sectional study of intensivists at public and private academic and nonacademic Latin American intensive care units (ICUs) through a web-based electronic survey submitted by email. Questions about personal aspects, work-related topics, and general clinical workflow were incorporated.

**Results:**

Our study comprised 735 survey respondents (53% return rate) with the following country-specific breakdown: Brazil (29%); Argentina (19%); Chile (17%); Uruguay (12%); Ecuador (9%); Mexico (7%); Colombia (5%); and Bolivia, Peru, Guatemala, and Paraguay combined (2%). Latin American intensivists were predominantly male (68%) young adults (median age, 40 [IQR, 35–48] years) with a median clinical ICU experience of 10 (IQR, 5–20) years. The median weekly workload was 60 (IQR, 47–70) h. ICU formal training was between 2 and 4 years. Only 63% of academic ICUs performed multidisciplinary rounds. Most intensivists (85%) reported adequate conditions to manage patients with septic shock in their units. Unsatisfactory conditions were attributed to insufficient technology (11%), laboratory support (5%), imaging resources (5%), and drug shortages (5%). Seventy percent of intensivists participated in research, and 54% read scientific studies regularly, whereas 32% read no more than one scientific study per month. Research grants and pharmaceutical sponsorship are unusual funding sources in Latin America. Although Latin American intensivists are mostly unsatisfied with their income (81%), only a minority (27%) considered changing to another specialty before retirement.

**Conclusions:**

Latin American intensivists constitute a predominantly young adult workforce, mostly formally trained, have a high workload, and most are interested in research. They are under important limitations owing to resource constraints and overt dissatisfaction. Latin America may be representative of other world areas with similar challenges for intensivists. Specific initiatives aimed at addressing these situations need to be devised to improve the quality of critical care delivery in Latin America.

**Electronic supplementary material:**

The online version of this article (10.1186/s13054-018-1956-6) contains supplementary material, which is available to authorized users.

## Background

Intensive care medicine (ICM) is a relatively young discipline that has rapidly grown into a full-fledged specialty. Given the aging of the population, the rising burden of chronic comorbidities, and the complexity of modern medicine, the intensive care unit (ICU) turns out to be the very last healthcare venue for many diseases. Intensivists are responsible for managing the ever-increasing number of patients with complex, life-threatening diseases [1]. Several factors could influence the performance of intensivists, including their age, training, experience, workload, and socioeconomic context. Latin America is a region that comprises mainly middle-income countries and deals with challenges such as poverty, low salaries, and low quality of employment [2]. We think it may well represent other world regions in terms of particular contexts and challenges that intensivists must overcome.

Although the commitment to provide high-quality patient care is firmly grounded in the medical profession, several recent publications have addressed noncompliance with best evidence practice by physicians [3]. This is perhaps most relevant for the treatment of patients with sepsis, a key area in critical care medicine in which several diagnostic and therapeutic approaches have been proposed over the years [4, 5]. To address some of these aspects, we developed a survey with the aim of gaining insight into how Latin American intensivists feel about some predetermined issues related to their daily work and expectations, as well as some aspects regarding the management of septic shock.

## Methods

This study was designed, coordinated, and executed by the Latin American Intensive Care Network - LIVEN (www.redliven.org) [6], which appointed a steering committee and local coordinators in each country.

### Design and setting

We conducted a cross-sectional study of intensivists from 11 Latin American countries (Argentina, Bolivia, Brazil, Chile, Colombia, Ecuador, Guatemala, Mexico, Paraguay, Peru, and Uruguay). The Pontificia Universidad Católica de Chile was the only coordinating center. The local ethics committee (Comité de Etica Clínica de la Facultad de Medicina) waived the need for informed consent because survey participation was voluntary; thus, informed consent is assumed.

In May 2016, Latin American intensivists, identified from lists provided by national critical care medicine societies and networks, social networks, and personal contacts, were invited to participate in the survey. One week later, a web-based electronic questionnaire was submitted. Weekly reminders were emailed to nonrespondents from June through September 2016.

### Questionnaire development

The survey was designed by a committee that incorporated questions regarding training, workload, competencies, continuing education, research activities, and experiential aspects. The construct was defined on the basis of studies that demonstrated links between individual characteristics of intensivists with clinical performance [7].

After a draft revision by a group of LIVEN investigators, some questions were reformulated, added, or deleted. Content validity was established by independent reviewers who determined whether each question captured the intended domain. After piloting the survey in 4 centers, the final version included 51 items under the following domains: organizational characteristics of the ICU, human resources, professional development, research participation, competencies and skills, satisfaction, and expectations.

We asked some questions about septic shock management because this condition integrates ICU workflow, resource availability, and diverse aspects of critical care provision. We considered that ICUs with appropriate conditions for septic shock care had availability of antibiotics, vasopressors, laboratory tests (arterial blood gases, serum lactate, general blood and biochemistry tests, blood and fluid cultures and microbiology identification), imaging resources, and the possibility to consult different specialists upon request (see full survey as Additional file 1: File S1).

We defined a long or short morning round on the basis of the duration being longer or shorter than 2 h, respectively. We defined a multidisciplinary round as one that, apart from the intensivists, included at least two other professionals (nurses, respiratory therapists, pharmacists, or other specialists).

### Data processing and statistical analysis

Data are expressed as mean ± SD or median (IQR), as appropriate. Categorical variables were compared with the chi-square test; continuous variables were analyzed with *t* tests, the Kruskal-Wallis test, and the Wilcoxon rank-sum test, according to their distribution. Logistic regression analysis was performed in a stepwise fashion according to individual covariates’ significance. ORs and 95% CIs were reported.

We investigated the association between country-level factors and relevant outcomes, adjusting for individuals’ characteristics using multilevel multivariable logistic regression. A two-level model was fit with intensivist-level fixed effects at the first level and country-level fixed effects at the second level, as well as a country-specific random effect. Individual-level variables of interest included age, sex, years of experience (collinear with age), weekly working time (in hours), and type of ICU (public/private, academic/nonacademic). We selected variables for the multivariable model using forward and backward stepwise regression. We considered variables for the model if they were associated with outcome with a *p* value less than 0.20 in univariate analysis. Additionally, some variables were introduced to the model because of their clinical relevance, regardless of their *p* value. We performed subgroup analyses by stratifying intensivists according to their position in the ICU. To choose among the alternative models, we used the likelihood ratio test for testing on the boundary of the parameter space as a measure of the relative predictive ability of a statistical model for a given set of data. Two-tailed *p* values less than 0.05 were considered statistically significant. We conducted all statistical analyses with the use of Stata 14.2 software (StataCorp, College Station, TX. USA).

## Results

### General

**Fig. 1:**
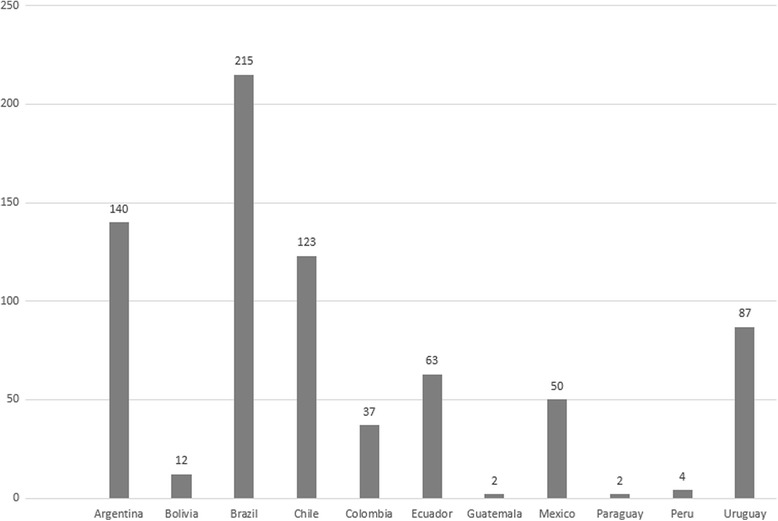
Number of surveys answered, by country

Of the 1380 surveys sent out, we received 735 responses by intensivists from 11 countries, yielding a global response rate of 53%. (See Fig. 1 for country representation.) Sixty percent of intensivists worked in ICUs located in public hospitals (Table 1, Additional file 2: Table S1A), with no statistically significant difference according to their position (Additional file 2: Table S1B). Sixty percent worked in two or more hospitals (Additional file 2: Table S1C, D), and 67% worked in academic hospitals (Table 1, Additional file 2: Table S1A), without any difference in the public/private status of their primary hospital (Additional file 2: Table S1D).

**Table 1:** General characteristics of study population

Variable	Data
Age, years, median (IQR)	40 (35–48)
Female sex, *n* (%)	238 (32%)
ICU experience, years, median (IQR)	10 (5–10)
ICU experience, *n* (%)	
< 10 years	391 (53)
11–20 years	197 (27)
21–30 years	123 (17)
> 30 years	24 (3)
ICU type (public)	440 (60)
ICU type (academic)	494 (67)
Primary specialty, *n* (%)	
Surgery	15 (2)
Emergency medicine	25 (3)
Anesthesia	31 (4)
Internal medicine only	136 (19)
Internal medicine	199 (27)
Intensive care medicine only	411 (56)
Intensive care medicine	505 (69)
Training, *n* (%)	
On-the-job training	177 (24)
2-year program	204 (28)
3-year program	152 (21)
4-year program	155 (21)
Other	45 (6)
No training	2 (0)
Reading scientific papers, *n* (%)	
Daily	43 (6)
1–3/week	346 (50)
1–2/month	305 (44)
Other	41 (6)

**Fig. 2:**
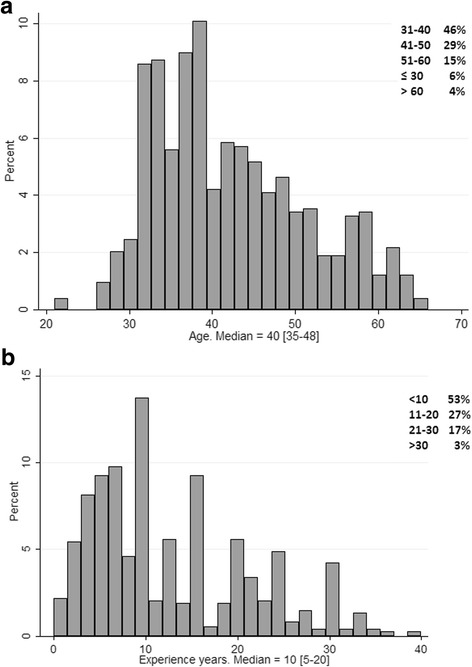
**a** Distribution of Latin American intensivists according to age. **b** Distribution of Latin American intensivists according to years of intensive care unit experience

The surveyed workforce was composed predominantly of males (68%) and young adults (median age, 40 [35–48] years) (Table 1, Fig. 2a). Fifty-three percent of intensivists had less than 10 years of experience in the ICU, and 20% had more than 20 years (Table 1, Fig. 2b). Residents comprised a minority of the respondents (13%).

ICU training varied among countries, with three different formal academic programs of variable duration (2–4 years) and a work-based assessment of competences without specific duration (Table 1, Additional file 3: Figure S1).

### Workload, clinical rounds, and competencies

**Fig. 3:**
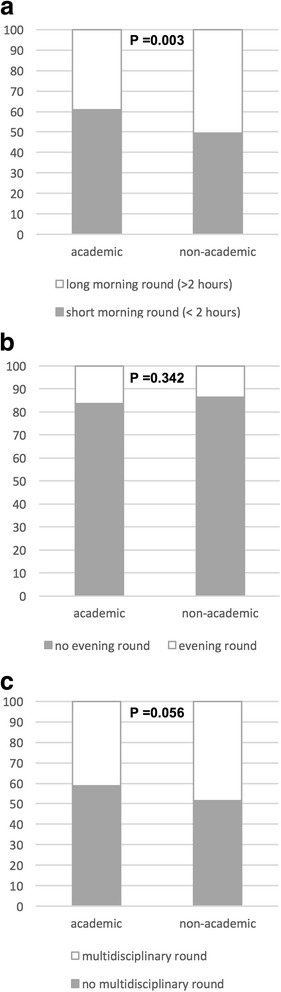
Proportion of intensive care unit rounds according to their academic status and (**a**) length of morning round, (**b**) presence of evening round, and (**c**) number of specialists involved in the morning round

The median weekly workload was 60 (IQR, 47–70) h, mostly spent in the ICU (Additional file 4: Figure S2), without differences between academic and nonacademic centers (median 60 [IQR, 46–70] vs. 60 [IQR, 48–68] h; *p* = 0.7). The duration and characteristics of clinical rounds varied widely across countries, with differences between academic and nonacademic units (Fig. 3).

Fifty-two percent of intensivists worked daily and also did night shifts. Twenty-two percent worked only in day shifts, and 22% only in night shifts. The median number of night shifts was 1 (IQR, 1–2) per week. Younger intensivists did more night shifts per week (*p* < 0.001). According to position, staff intensivists performed the higher number of shifts per week (median, 2 [IQR, 1–3]), followed by residents (median, 2 [IQR, 1–2]) (*p* < 0.001). The number of beds covered per intensivist varied from 3 to 12 per night shift. Most ICUs (76%) had consultants available during night shifts. Overall, survey respondents considered themselves very competent to perform many common procedures. There were significant differences according to position, age, and years of experience in the ICU (Table 2).

**Table 2:** Self-reported confidence in competency to perform clinical duties, according to intensivist position, age, and years of experience

Procedure		ICU director	Medical coordinator	Staff physicians	Residents	Shift physicians	Other	Overall	*p* Value
Intensivists by position									
Intubation	Yes	122 (98)	104 (100)	362 (100)	91 (95)	35 (100)	12 (92)	726 (99)	<0.001
	No	2 (2)	0 (0)	0 (0)	5 (5)	0 (0)	1 (8)	8 (1)	
Central venous catheter insertion	Yes	124 (100)	104 (100)	359 (99)	92 (96)	35 (100)	12 (92)	726 (99)	0.005
	No	0 (0)	0 (0)	3 (1)	4 (4)	0 (0)	1 (8)	8 (1)	
Arterial line insertion	Yes	120 (97)	103 (99)	350 (97)	89 (93)	35 (100)	10 (77)	707 (96)	0.001
	No	4 (3)	1 (1)	12 (3)	7 (7)	0 (0)	3 (23)	27 (4)	
PA catheter placement	Yes	111 (90)	90 (87)	290 (80)	66 (69)	26 (74)	7 (54)	590 (80)	<0.001
	No	13 (10)	14 (14)	72 (20)	30 (31)	9 (26)	6 (46)	144 (20)	
Echocardiography	Yes	53 (43)	43 (41)	149 (41)	42 (44)	11 (31)	3 (23)	301 (41)	0.621
	No	71 (57)	61 (59)	213 (59)	54 (56)	24 (69)	10 (77)	433 (59)	
End-of-life care issues management	Yes	122 (98)	101 (97)	337 (93)	78 (81)	30 (86)	10 (77)	678 (92)	<0.001
	No	2 (2)	3 (3)	25 (7)	18 (19)	5 (14)	3 (23)	56 (8)	
Intensivists by age									
Intubation	Yes	45 (41–51)	44 (38–52)	39 (35–46)	33 (31–38)	40 (35–53)	39 (37–46)	40 (35–48)	<0.001
	No	52 (41–63)	–	–	31 (31–33)	–	31 (31–31)	32 (31–39)	0.202
Central venous catheter insertion	Yes	45 (41–51)	44 (38–52)	39 (35–46)	33 (31–38)	40 (35–53)	39 (37–46)	40 (35–48)	<0.001
	No	–	–	34 (29–37)	32 (31–35)	–	31 (31–31)	32 (31–35)	0.513
Arterial line insertion	Yes	45 (41–51)	44 (38–52)	39 (35–46)	33 (31–38)	40 (35–53)	38 (37–47)	40 (35–48)	<0.001
	No	49 (44–54)	47 (47–47)	36 (33–53)	32 (30–33)	–	39 (31–42)	35 (32–47)	<0.001
PA catheter placement	Yes	45 (41–51)	43 (38–51)	40 (35–47)	34 (31–39)	41 (34–53)	44 (37–48)	41 (35–49)	<0.001
	No	44 (41–60)	45 (39–52)	36 (33–42)	32 (29–34)	40 (39–45)	35 (32–39)	37 (32–45)	<0.001
Echocardiography	Yes	46 (40–51)	39 (37–48)	38 (34–44)	34 (31–37)	39 (38–40)	39 (37–39)	39 (34–45)	<0.001
	No	45 (42–52)	47 (41–54)	40 (35–49)	33 (31–38)	45 (34–53)	40 (33–47)	42 (35–50)	<0.001
End-of-life care issues management	Yes	46 (41–51)	45 (38–52)	39 (35–46)	33 (31–38)	42 (38–53)	41 (37–47)	40 (35–49)	<0.001
	No	39 (36–41)	38 (37–41)	38 (35–42)	31 (30–37)	37 (33–44)	33 (31–37)	36 (31–41)	0.092
Intensivists by years of experience									
Intubation	Yes	16 (10–23)	15 (10–24)	10 (5–16)	4 (2–6)	12 (7–20)	10 (5–12)	10 (6–20)	<0.0001
	No	22 (9–34)	–	–	2 (2–4)	–	2 (2–2)	3 (2–8)	0.1193
Central venous catheter insertion	Yes	16 (10–23)	15 (10–24)	10 (5–17)	4 (2–6)	12 (7–20)	10 (5–12)	10 (6–20)	<0.0001
	No	–	–	2 (1–7)	2 (1–4)	–	2 (2–2)	2 (2–4)	0.9463
Arterial line insertion	Yes	15 (10–23)	15 (10–24)	10 (6–16)	4 (2–6)	12 (7–20)	10 (5–10)	10 (6–20)	<0.0001
	No	21 (18–24)	12 (12–12)	6 (2–19)	2 (1–6)	–	5 (2–14)	3 (2–16)	0.0315
PA catheter placement	Yes	16 (10–23)	15 (10–24)	10 (6–20)	5 (2–7)	12 (6–21)	10 (10–14)	11 (6–20)	<0.0001
	No	12 (9–27)	12 (11–22)	7 (4–10)	2 (1–5)	12 (9–12)	5 (4–8)	7 (4–12)	<0.0001
Echocardiography	Yes	15 (10–22)	10 (9–16)	9 (5–15)	4 (2–6)	10 (6–12)	8 (5–14)	10 (5–16)	<0.0001
	No	16 (10–24)	20 (12–25)	10 (6–20)	3 (2–5)	14 (8–22)	10 (5–10)	11 (6–20)	<0.0001
End-of-life care issues management	Yes	16 (10–23)	15 (10–24)	10 (6–18)	4 (2–6)	12 (7–20)	10 (5–14)	16 (10–20)	<0.0001
	No	9 (8–10)	10 (8–10)	5 (5–12)	2 (1–6)	10 (8–14)	4 (2–8)	5 (2–10)	0.0106

*ICU* Intensive care unit, *PA* Pulmonary artery

*p* Values for intensivists by position are for yes/no proportions. Age and years of experience are expressed as median (IQR)

### Septic shock management

Most intensivists (85%) reported adequate conditions to manage patients with septic shock. However, perception of satisfactory conditions was lower in ICUs located in public hospitals (OR, 0.36 [0.26–0.47]; *p* < 0.001) and in nonacademic ICUs (OR, 0.48 [0.35–0.65]; *p* < 0.001). The main reasons for dissatisfaction were attributed to insufficient technology (11%), laboratory support (5%), imaging resources (5%), and limited drug availability (5%). Lactate could be measured by 90% of intensivists, but at a variable time, depending on ICU type (Additional file 5: Table S2A). A computed tomographic (CT) scan was available within < 2 h in 54% of ICUs and within < 6 h in 81% of ICUs, with variations according to ICU type (Additional file 5: Table S2B). In all countries, ICUs located in private hospitals had significantly greater availability of CT scanners compared with ICUs belonging to the public health sector (68% vs 49% in < 2 h; OR, 3.13 [2.02–5.94]; *p* < 0.001).

### Education and research

The Internet was widely accessible (91%). Most Latin American intensivists used online resources to improve their scientific knowledge (96%). More than half of the intensivists surveyed (54%) read scientific studies regularly, daily, or two or three times per week (Table 2), whereas 32% read no more than one scientific study per month. These results were similar for academic and nonacademic ICUs (*p* = 0.6). Seventy-five percent of respondents had attended at least one scientific meeting in the past year, and 88% had taken a refresher course. Seventy-six percent reported having “some” or “much” institutional support, but 24% responded that they had “minimal” or “no support” from their hospitals to participate in these meetings. Financial and permission restrictions were the main reasons (Table 3).

**Table 3:** Institutional restrictions on attending scientific meetings in participating countries

Restrictions		No.	%		
Some		262	36		
Much		299	41		
None		72	10		
Few		102	14		
Total		735	100		
Country	Financial restrictions	Permission restrictions	Time restrictions	Other	Total
Argentina	66 (68)	18 (19)	11 (11)	2 (2)	97 (100)
Bolivia	4 (36)	6 (55)	1 (9)	0 (0)	11 (100)
Brazil	49 (36)	58 (44)	0 (0)	26 (20)	133 (100)
Chile	14 (25)	39 (70)	2 (4)	1 (2)	56 (100)
Colombia	8 (35)	13 (57)	2 (9)	0 (0)	23 (100)
Ecuador	23 (40)	33 (57)	1 (2)	1 (2)	58 (100)
Guatemala	2 (100)	0 (0)	0 (0)	0 (0)	2 (100)
Mexico	8 (24)	23 (70)	2 (6)	0 (0)	33 (100)
Paraguay	0 (0)	1 (100)	0 (0)	0 (0)	1 (100)
Peru	1 (100)	0 (0)	0 (0)	0 (0)	1 (100)
Uruguay	28 (49)	19 (33)	9 (16)	1 (2)	57 (100)
Overall *n* (%)	203 (43)	210 (45)	28 (5)	31 (7)	472 (100)

Permission restrictions refer to nonauthorization to attend a meeting owing to managerial reasons

Seventy percent of the respondents participated in research activities, mostly in academic settings (71%). Working in a nonacademic ICU and being a resident were associated with less probability of doing research (OR, 0.50 [0.36–0.73]; and OR, 0.36 [0.23–0.58], respectively; *p* < 0.001 for both). Conversely, being male was associated with higher probability of doing research (OR, 1.52 [1.08–2.15]; *p* = 0.017). Most of the funding came from intensivists’ respective institutions (39%) or was self-provided (19%), whereas 14% of intensivists declared no specific research funding. Research grants and pharmaceutical sponsorship were rarely available (9% and 7%, respectively). Most of the research was published in peer-reviewed journals (63%).

### Perceptions and expectations

Environmental conditions (private restrooms, comfortable bedrooms for rest or sleep) during night shifts were reported as inappropriate in 62% of the respondents, especially by female intensivists (71% vs. 60%; *p* < 0.001). A minority of intensivists were satisfied with their personal income (19%). It was higher in male than in female physicians (22% vs 13%; OR, 1.84 [1.17–2.91]; *p* = 0.009). On the contrary, higher weekly workload (OR, 0.98 [0.97–0.99]; *p* = 0.017) and a higher number of night shifts (OR, 0.80 [0.67–0.96]; *p* = 0.007) were associated with lower income satisfaction. Overall, a minority of intensivists (27%) reported having considered quitting their job as intensivists, mostly in Argentina (41%) and Brazil (36%). Fifty-five percent on the Latin American intensivists intended to leave ICU practice before retirement.

## Discussion

This is the first study in Latin America involving evaluation of individual- and work-related aspects of the intensivist workforce. Our main findings were that the intensivist workforce of the region, mostly related to academic centers, is predominantly young, has been formally trained, report adequate procedural skills, and operates under high workloads and restrictions owing to resource constraints and local limitations. Although there is no ideal percentage, a higher response rate to our questionnaire would have been desirable. However, our first survey (LIVEN-1) had a similar return rate (52%) [6], which is in line with the declining response rates to surveys over the years [8, 9].

Health systems vary across Latin American countries, and a mix of public and private ICUs do exist, sometimes with uneven resource distribution [10, 11]. In our study, most intensivists worked primarily in public hospitals, but less than half worked in only one hospital. This could have an impact in terms of costs and efficiency because working in more than one hospital might result in higher costs owing to “work dispersion” [12].

Our respondents reported having mostly formal ICM training (70%), mainly under the supraspecialty model, which considers training in a base specialty followed by a common ICM program. This was also the most frequent training mechanism in a 41-country survey published some time ago [13]. Other mechanisms, such as the assessment of competencies, are common in Chile and Brazil, perhaps in response to the shortage of intensivists [14, 15].

With regard to workload, working more than 60 h per week was associated with a high level of burnout in a recent study [16]. Intention to leave is a known predictor of burnout [17, 18], as well. Because 60 h was the median weekly workload of our respondents and more than half of them reported their desire to leave the ICU before retirement, Latin American academic intensivists probably experience high-level burnout. Along the same line, a recent study of ICM training program directors showed that higher workload correlated with negative self-perception about the teaching role, patient care, and job stability [19]. In addition, the number of night shifts has clearly been associated with burnout among pediatric intensivists [20] and critical care nurses [21]. In our survey, staff physicians reported a higher night shift load and higher intention to leave, an association described previously [20, 21]. These findings call for responsible authorities to be concerned about intensivists’ workload and mental health.

Our finding that short and non-multidisciplinary morning rounds occur mainly in academic ICUs, unveiling the tensions that academic intensivists may experience in performing high-quality clinical work in time- and resource-restricted contexts. Conversely, those in many nonacademic ICUs performed long morning rounds, a fact not easily reconciled with the previous one. Academic medical centers share a mission of patient care, teaching, and research [22], but financial pressures might promote the former to the detriment of teaching and research [22] despite the “academic” denomination [23]. This could be happening in Latin America, where financial challenges [2] compete with education at all levels.

Procedural complications are a significant cause of inpatient morbidity and mortality in the ICU [24]. In our surveyed population, self-perception of technical skills was high, especially among older and more experienced doctors. Residents tended to exhibit lower self-confidence in most procedures, but they were a minority. Our results reflect the well-known progression in skill levels after training and years of experience [25].

Regarding septic shock management, some intensivists reported insufficient conditions to treat it adequately, mentioning drug shortages, among other reasons. Increased mortality has been observed during shortages of drugs in low-income [26, 27] and high-income countries [28]. High mortality of sepsis and septic shock reported in Latin America [27, 29] could be partially explained by this.

Additionally, we considered CT scans and lactate measurements as proxies for clinical workflow and resource availability. Lactate measurement availability was acceptable overall, with some differences in public vs private hospitals. On the contrary, in private ICUs, CT scanners were much more accessible. In fact, although we did not study the relationship of these resources with any outcomes, it has been demonstrated that resource inequality is a determinant of quality of care [30] and health outcomes in the ICU [31, 32], especially in resource-poor settings.

Latin American intensivists preferred online resources as the source of scientific information, similar to U.S. physicians [33]. Among our respondents, the rate of reading scientific studies was lower than reported in other studies [34]. Because ICU academic status was not a determinant for scientific reading, knowledge acquisition seems to rely on personal interests. Most intensivists would have attended scientific meetings outside their hospitals, but they were hindered by financial and permission restrictions. This contrasts with a study involving physicians from high-income countries [35], in which researchers reported attendance at a considerable number of meetings each year. The same study showed that congresses and conferences are preferred, which is similar to our results.

Barriers to participation in clinical research in developing countries are widely known [36]. Two-thirds of surveyed physicians showed interest in research and published some work in a peer-reviewed journal. A study on physicians from different specialties, excluding intensivists, yielded similar results, showing that 63% of them had published articles in medical journals [34]. Our reported participation in research is high, probably owing to the academic connections of our respondents.

End-of-life care is an area of increasing prominence in the ICU [37], but studies have shown that, for example, appropriate relief of suffering and pain in dying patients is dissimilar in ICUs [38]. In this field, Latin America presents regional shortcomings related to inadequate legislation, insufficient infrastructure, lack of opportunities for clinical training, unreliable reporting of data, and cultural barriers [39]. Younger intensivists reported lower confidence than their older and more experienced colleagues in addressing these issues. A leveraging agenda must be developed to provide all intensivists with the competencies required to address these patients’ needs properly.

Job satisfaction is a multidomain perception related to many factors [40]. We did not address it specifically but instead asked about income, which has been related to general and emotional well-being as well as job satisfaction [40, 41]. In our study, most respondents considered their income unsatisfactory, especially female and middle-aged physicians. In a recent study in Latin America, being female was associated with lower job satisfaction as well as higher workload [42]. How these issues interact with the expectations, rewards, and drawbacks of working as an intensivist still need to be more completely elucidated.

Our study has several limitations. The results are not generalizable to all Latin American countries. This study was performed with a convenience sample of physicians working in Latin American ICUs, predominantly academic, with respondents probably more prone to read scientific literature and to conduct research. The heading of the survey questions asked that respondents answer thinking about the ICU where they work most hours per week, but undoubtedly this could have introduced bias. We did not interrogate for burnout, moral distress, specific end-of-life care issues, or communication and management skills. In-training physicians were underrepresented in this sample, as were physicians working in ICUs without board certification. Regardless of these considerations, in the absence of previous information, this is the first description of general, individual-, and work-related characteristics of the intensivist workforce in Latin America, mainly at academic ICUs.

## Conclusions

Latin American intensivists are still a young adult group of physicians with unique problems that include a high weekly workload, important resource constraints, job dissatisfaction, and financial limitations on and barriers to attending educational opportunities. Many challenges remain unsettled in terms of training and competencies to develop, as well as how to achieve workload balance. Some of the issues described may help to depict the panorama of the delivery of critical care around the world.

## Additional files

Additional file 1: File S1. LIVEN SHOCK II- Physicians Survey. (PDF 339 kb)

Additional file 2: Table S1. Distribution of: (A) academic vs non-academic ICUs according to their public or private status, (B) ICU condition (public/private) where intensivists work, by role, (C) number of ICUs where intensivists work by role, and (D) number of different ICU where intensivists work according to public or private intensivists’ primary hospital. (DOCX 87 kb)

Additional file 3: Table S2. Intensive care medicine training programs duration in LIVEN-2 respondents according to country. (TIFF 6093 kb)

Additional file 4: Figure S1. Proportion of weekly workload spent in the ICU versus non-ICU work of LIVEN-2 respondents according to country. (TIFF 6207 kb)

Additional file 5: Figure S2. Availability of: (A) lactate and (B) CT-scan, according to ICU-type and time. (DOCX 59 kb)

## Abbreviations

CT: Computed tomographic
ICM: Intensive care medicine
ICU: Intensive care unit
LIVEN: Latin American Intensive Care Network
PA: Pulmonary artery
