# Supplementary material for: The practice of intensive care in Latin America: a survey of academic intensivists
Source: Crit Care. 2018 Feb 21;22:39. doi: 10.1186/s13054-018-1956-6 (PMC5820791; doi:10.1186/s13054-018-1956-6)
Supplement: Supplementary file 1 — LIVEN SHOCK II- Physicians Survey. (PDF 339 kb) [file 13054_2018_1956_MOESM1_ESM.pdf]

LIVEN SHOCK II- Physicians Survey

Background

El Shock es una disfunción circulatoria grave y de elevada mortalidad, constituyendo una de las principales causas de ingreso a las Unidades de Cuidado Intensivo (UCI).

La sobrevida de esta condición depende de varios factores pero uno muy crítico es la disponibilidad de recursos humanos y técnicos adecuados. En lo humano esto implica la cantidad y calidad (en el sentido formativo) del equipo médico, de enfermería y profesiones afines que trata al paciente. En lo técnico están involucrados muchos factores incluyendo aspectos de monitoreo, drogas, laboratorio, entre otros.

No existe información sobre estos aspectos en Latinoamérica en la literatura y un primer paso hacia un cambio positivo es siempre el conocer la realidad a través de estudios como este.

Propósito de la Encuesta

Obtener información sobre los médicos que atienden a los pacientes en shock en las UCIs de Latinoamérica con énfasis en aspectos formativos, humanos, carga de trabajo y percepción de capacidades.

LIVEN SHOCK II- Physicians Survey

1. País

2. Ciudad

[ ]

3. Sexo

- ○ Hombre
- ○ Mujer

4. ¿Cuál es su edad?

[ ]

5. ¿Cuál es su especialidad primaria?

- ☐ Anestesia
- ☐ Medicina interna
- ☐ Cirugía
- ☐ Medicina de urgencia
- ☐ Medicina intensiva
- ☐ Otro (especifique)

[ ]

6. Aproximadamente, ¿cuántos años de experiencia en UCI tiene Ud.?

[ ]

7. ¿Cómo se formó Ud. como intensivista?

- ○ Entrenamiento en práctica
- ○ Programa formal de 2 años
- ○ Programa formal de 3 años
- ○ Programa formal de 4 años
- ○ Otro (especifique)

[ ]

8. ¿Ud. trabaja en el mismo país en donde realizó su formación médica?

- ○ Sí
- ○ No

9. Aproximadamente, ¿qué porcentaje de su jornada laboral semanal la realiza en UCI?

- 25% o menos
- 26 a 50%
- 51 a 75%
- 75% o más

10. Aproximadamente, ¿cuántas horas semanales trabaja Ud. en promedio?

11. ¿Qué jornada de trabajo realiza Ud. en UCI?

- Solo guardias
- Trabajo diurno diario y guardias
- Solo trabajo diurno
- Otro (especifique)

12. Aproximadamente, ¿cuántas horas a la semana trabaja Ud. en UCI?

13. ¿Cuántas guardias realiza Ud. a la semana?

14. ¿En cuantos centros trabaja Ud.?

- 1 centro
- 2 centros
- 3 centros
- Otro (especifique)

15. La UCI donde Ud. trabaja es,

	Tipo de UCI I	Tipo de UCI II	Tipo de UCI III
UCI			

16. ¿Cuántas camas de UCI tiene a su cargo en una guardia habitual?

17. En la UCI donde Ud. trabaja la mayor cantidad de horas,

¿Existe entrega de turno formal?

¿Cuánto tiempo tarda la entrega de turno?

¿Existe un documento estructurado para la entrega de turno?

18. En la UCI donde Ud. trabaja la mayor cantidad horas a la semana, las rondas son:

	Si	No
Multidisciplinarias	○	○
Ronda matinal extensa	○	○
Ronda matinal académica	○	○
Ronda por las tardes	○	○

19. En la UCI donde Ud. trabaja la mayor cantidad de horas semanales, las(os) enfermeras(os) tienen formación:

- ○ Universitaria
- ○ Técnica
- ○ Mezcla de universitaria / técnica
- ○ ¿Cuantos años de formación tienen las enfermeras?

20. En su unidad, ¿con qué frecuencia asiste a reuniones clínicas?

- ○ Mensual
- ○ Cada 2 semanas
- ○ Semanal
- ○ Nunca
- ○ Otro (especifique)

21. ¿Ud. ha participado en investigación clínica?

- Sí
- No

22. Si su respuesta anterior es afirmativa, ¿cuál fue el producto de su trabajo?

	Sí	No
Publicación en revista científica		
Presentación en congreso (presentación oral o póster)		

Fuente de financiamiento, especifique (institucional, fondo concursable, laboratorio, otro)

23. ¿Asistió a congresos de la especialidad de medicina intensiva el último año?

- Sí
- No

24. ¿Ud. realizó el ACLS o ATLS en los últimos 5 años?

- Sí
- No

25. ¿Ud. realizó cursos de actualización en el último año?

- Sí
- No

26. ¿Ud. piensa tomar algún curso de actualización en los próximos dos años?

- Sí
- No

27. En su opinión, ¿qué facilidades tiene Ud. para perfeccionamiento y asistencia a cursos o congresos?

- Muchas facilidades
- Escasas facilidades
- Ninguna facilidad

28. Si en su opinión, Ud tiene escasas o ninguna facilidad para perfeccionarse, ¿a qué lo atribuye?

- ◯ Restricción a los permisos
- ◯ Limitaciones económicas
- ◯ Otro (especifique)

29. En general, ¿Ud. lee regularmente artículos científicos?

- ◯ Sí
- ◯ No

30. Si su respuesta anterior es afirmativa, ¿Con qué regularidad lo hace?

- ◯ Una vez por semana
- ◯ Una vez al mes
- ◯ Dos veces al mes
- ◯ Todos los días
- ◯ Otro (especifique)

31. ¿Cómo actualiza sus conocimientos en la especialidad?

- ☐ Acceso sitios públicos de información
- ☐ Acceso online a principales revistas científicas de la especialidad
- ☐ Entrega de material por los laboratorios
- ☐ Congresos/cursos
- ☐ Otro (especifique)

32. En general, ¿Ud. confía en la información que los laboratorios le entregan?

- ◯ Sí
- ◯ No

33. En general, ¿Ud. considera que posee las competencias necesarias para los siguientes escenarios?

	Sí	No
Intubación de pacientes	◯	◯
Instalación de línea arterial	◯	◯
Instalación de catéter venoso central	◯	◯
Instalación de catéter de arteria pulmonar	◯	◯
Realizar ecocardiografía básica	◯	◯
Tomar decisiones de fin de vida	◯	◯

34. En su opinión, en relación al shock séptico, ¿en su unidad están las condiciones adecuadas para un manejo correcto?

- ◯ Sí
- ◯ No

35. Si su respuesta anterior es negativa, ¿a qué lo atribuye Ud.?

- ◯ Recursos tecnológicos insuficientes (dispositivos, monitores, catéteres, etc)
- ◯ Apoyo de imágenes insuficiente (poca disponibilidad y complejidad)
- ◯ Apoyo insuficiente de especialistas
- ◯ Disponibilidad de drogas y antibióticos insuficiente
- ◯ Apoyo de laboratorio insuficiente
- ◯ Recursos humanos insuficientes (médicos, enfermeras, etc)
- ◯ Otro (especifique)

36. En general, ¿qué nivel de satisfacción tiene Ud. con el manejo de los pacientes con shock séptico en su unidad?

- ◯ Muy satisfecho
- ◯ Medianamente satisfecho, hay cosas por mejorar y dependen de mi
- ◯ Medianamente satisfecho, hay cosas por mejorar que no dependen de mi
- ◯ Insatisfecho

37. En su institución, cuando Ud. solicita una TAC de urgencia, el examen se realiza:

- ◯ Antes de 2 horas
- ◯ Entre 2 a 6 horas
- ◯ En el día
- ◯ Al día siguiente
- ◯ No es seguro cuanto tiempo demorará en realizarse el examen

38. En su institución, cuando Ud. solicita un lactato de urgencia, el examen se realiza:

- ◯ Antes de 2 horas
- ◯ Entre 2 a 6 horas
- ◯ En el día
- ◯ No está disponible en mi institución

39. ¿Ha pensado alguna vez en abandonar la especialidad?

- ◯ Sí
- ◯ No

40. En relación a su calidad de vida como intensivista, Ud:

	Sí	No
Cree que sus remuneraciones son adecuadas para su responsabilidad y carga de trabajo	◯	◯
Considera que las condiciones ambientales en las guardias son adecuadas	◯	◯
Tiene apoyo de pares con más experiencia	◯	◯
Puede tomar vacaciones regularmente	◯	◯
Tiene acceso a internet en las guardias	◯	◯

41. ¿Como proyecta su carrera profesional?

- ◯ Ser intensivista hasta mi retiro
- ◯ Dejar la especialidad en los próximos 5 años
- ◯ Dejar guardias nocturnas y mantener cargo diurno en la especialidad
- ◯ Otro (especifique)
