# Supplementary material for: The practice of intensive care in Latin America: a survey of academic intensivists
Source: Crit Care. 2018 Feb 21;22:39. doi: 10.1186/s13054-018-1956-6 (PMC5820791; doi:10.1186/s13054-018-1956-6)
Supplement: Supplementary file 2 — Distribution of: (A) academic vs non-academic ICUs according to their public or private status, (B) ICU condition (public/private) where intensivists work, by role, (C) number of ICUs where intensivists work by role, and (D) number of different ICU where intensivists work according to public or private intensivists’ primary hospital. (DOCX 87 kb) [file 13054_2018_1956_MOESM2_ESM.docx]

**eTable 1**

1. Distribution of academic vs non-academic ICUs our study, according to their public or private status.
2. Distribution of ICU condition (public/private) where intensivists work, by role
3. Distribution of number of ICU where intensivists work, by role.
4. Distribution of number of different ICU where intensivists work, according to public or private intensivists’ primary hospital.

A

| **ICU type** | **Academic** | **Non-academic** | **Total** |
| --- | --- | --- | --- |
|  | n(%) |  |  |
| Private n (%) | 165 (56) | 130 (44) | 295 (100) |
| Public n (%) | 329 (75) | 111 (25) | 440 (100) |
|  |  |  |  |
| Total | 494 (67) | 241 (33) | 735 (100) |

P = 0.000

B

| **ICU condition** | **Intensivist role** | | | | | | **Total** |
| --- | --- | --- | --- | --- | --- | --- | --- |
|  | Medical coordinator | Other | Resident | Staff physician | Shift physician | ICU director |  |
|  | n(%) |  |  |  |  |  |  |
| Private | 55 (53) | 3 (23) | 38 (40) | 133 (37) | 16 (46) | 50 (40) | 295 (40) |
|  |  |  |  |  |  |  |  |
| Public | 49 (47) | 10 (77) | 58 (60) | 229 (63) | 19 (54) | 74 (60) | 439 (60) |
|  |  |  |  |  |  |  |  |
| Total | 104 | 13 | 96 | 362 | 35 | 124 | 734 |
|  | n(%) |  |  |  |  |  |  |

P = 0.055

| C |  |  |  |  |  |  |  |
| --- | --- | --- | --- | --- | --- | --- | --- |
| **Number different ICUs where intensivists work** | **Intensivist role** | | | | | | **Total** |
|  | Medical coordinator | Other | Resident | Staff physician | Shift physician | ICU director |  |
|  | n(%) |  |  |  |  |  |  |
| 1 | 32 (31) | 7 (54) | 54 (56) | 128 (36) | 7 (20) | 66 (53) | 294 (40) |
|  |  |  |  |  |  |  |  |
| 2 | 54 (53) | 5 (38) | 31 (32) | 145 (41) | 19 (54) | 49 (40) | 303 (42) |
|  |  |  |  |  |  |  |  |
| 3 | 16 (16) | 1 (8) | 11 (11) | 72 (20) | 6 (17) | 9 (7) | 115 (16) |
|  |  |  |  |  |  |  |  |
| 4 | 0 (0) | 0 (0) | 0 (0) | 4 (1) | 3 (9) | 0 (0) | 7 (1) |
|  |  |  |  |  |  |  |  |
| 5 | 0 (0) | 0 (0) | 0 (0) | 9 (3) | 0 (0) | 0 (0) | 9 (1) |
|  |  |  |  |  |  |  |  |
| Total | 102 | 13 | 96 | 358 | 35 | 124 | 728 |
|  |  |  |  |  |  |  |  |

P = <0.001

D

| **Number different ICUs where intensivists work** | **Type of ICU where intensivists predominantly belong** | | **Total** |
| --- | --- | --- | --- |
|  | Public | Private |  |
|  | n(%) |  |  |
| 1 | 170 (58) | 124 (42) | 294 (100) |
|  |  |  |  |
| 2 | 187 (62) | 117 (38) | 304 (100) |
|  |  |  |  |
| 3 | 67 (58) | 48 (42) | 115 (100) |
|  |  |  |  |
| 4 | 4 (57) | 3 (43) | 7 (100) |
|  |  |  |  |
| 5 | 9 (100) | 0 (0) | 9 (100) |
|  |  |  |  |
| **Total** | 57 (60) | 292 (40) | 729 (100) |

P = 0.095
