# Supplementary figures and images for: The practice of intensive care in Latin America: a survey of academic intensivists
Source: Crit Care. 2018 Feb 21;22:39. doi: 10.1186/s13054-018-1956-6 (PMC5820791; doi:10.1186/s13054-018-1956-6)

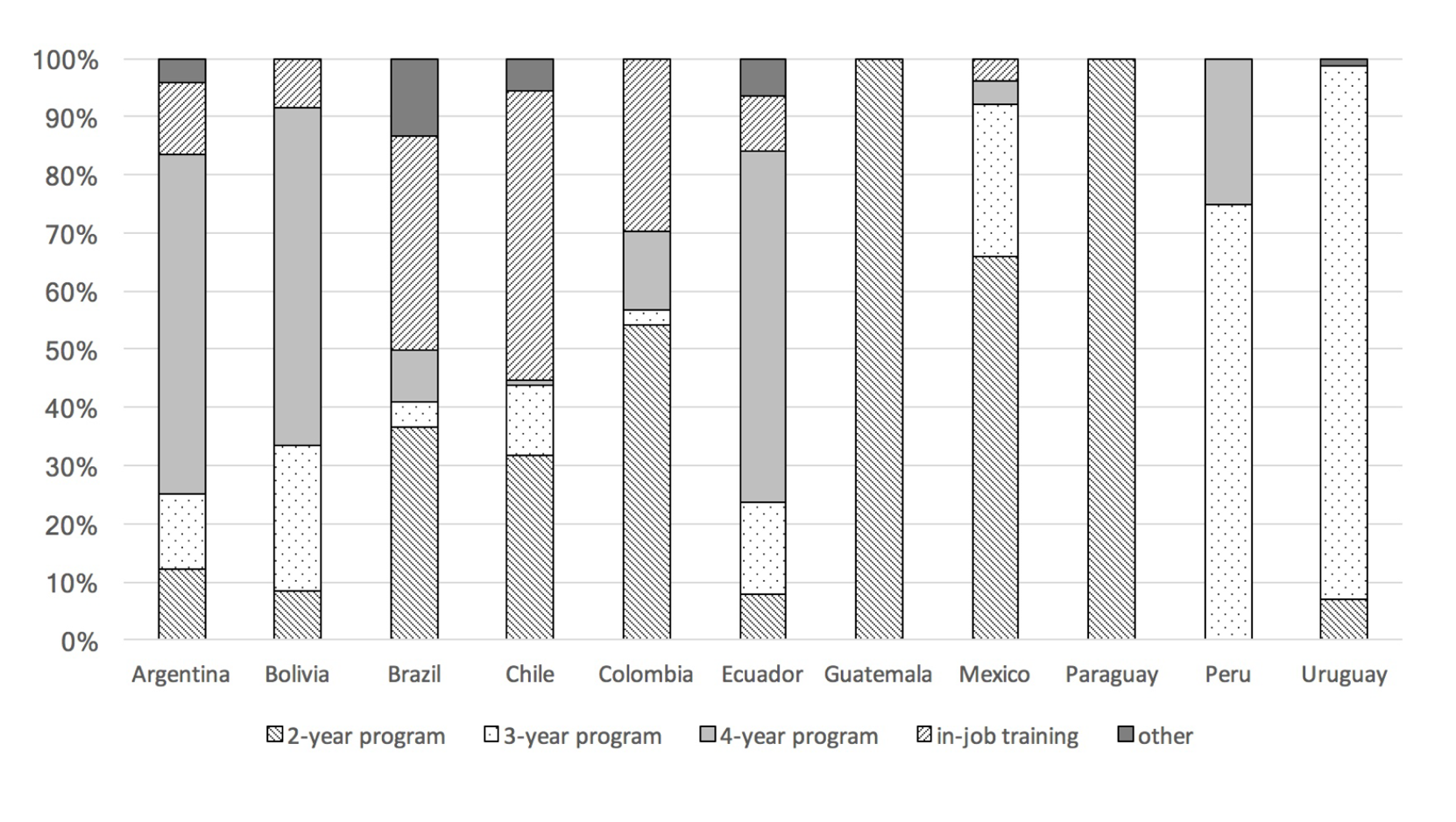

Supplement: Supplementary file 3 — Intensive care medicine training programs duration in LIVEN-2 respondents according to country. (TIFF 6093 kb) [file 13054_2018_1956_MOESM3_ESM.tiff]

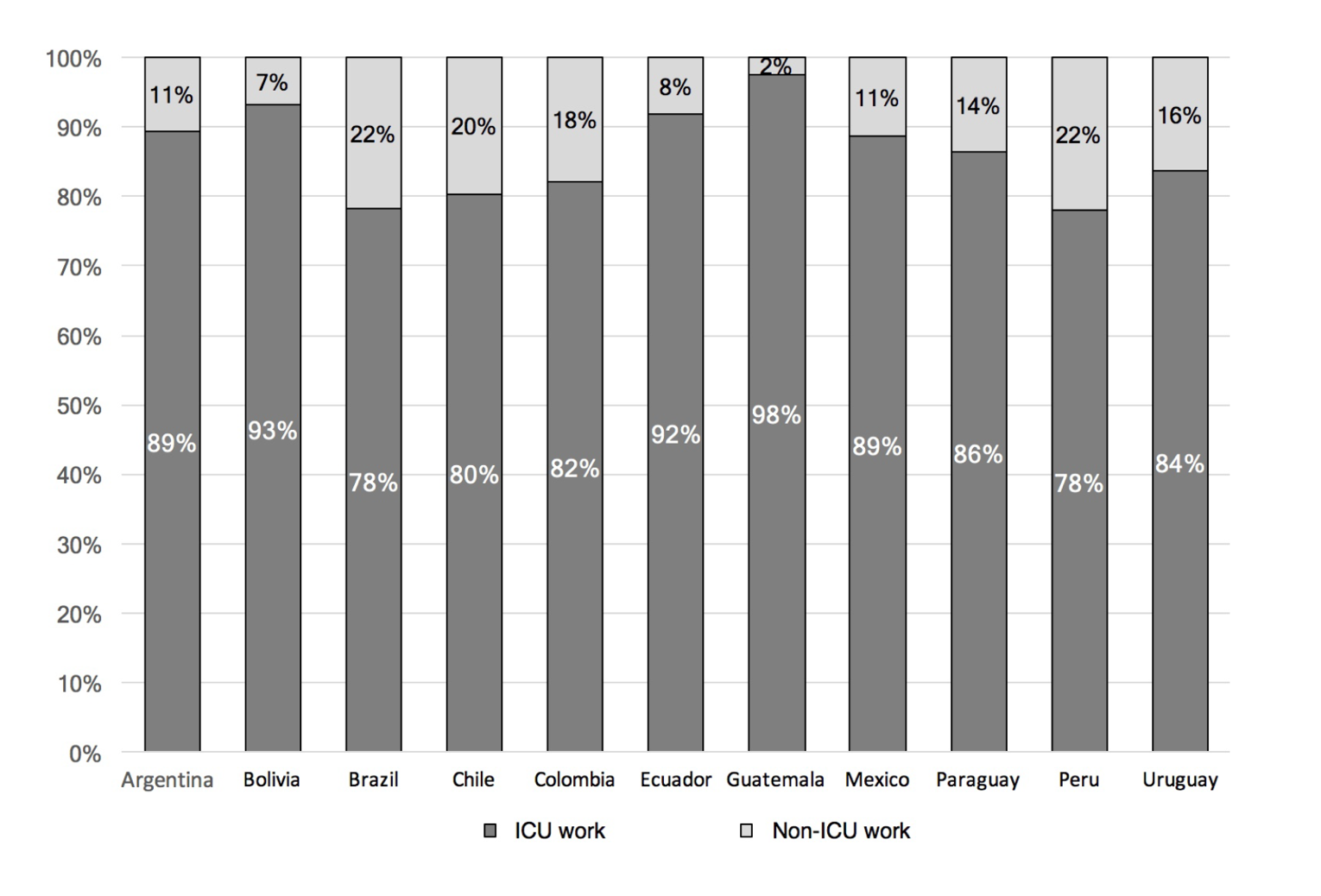

Supplement: Supplementary file 4 — Proportion of weekly workload spent in the ICU versus non-ICU work of LIVEN-2 respondents according to country. (TIFF 6207 kb) [file 13054_2018_1956_MOESM4_ESM.tiff]
