# Supplementary material for: The practice of intensive care in Latin America: a survey of academic intensivists
Source: Crit Care. 2018 Feb 21;22:39. doi: 10.1186/s13054-018-1956-6 (PMC5820791; doi:10.1186/s13054-018-1956-6)
Supplement: Supplementary file 5 — Availability of: (A) lactate and (B) CT-scan, according to ICU-type and time. (DOCX 59 kb) [file 13054_2018_1956_MOESM5_ESM.docx]

**eTable 2**

A. Lactate availability according to ICU-type and time. B. CT-scan availability according to ICU-type and time.

A

| **Lactate availability** | **ICU type** | | **Total** |
| --- | --- | --- | --- |
|  | Public | Private |  |
|  | n (%) |  |  |
| <2 h | 252 (80) | 177 (87) | 429 (83) |
| 2-6 h | 14 (4) | 14(7) | 28 (5) |
| >6 h | 8 (3) | 1(0) | 9 (2) |
| Unavailable | 43 (14) | 11(5) | 54 (10) |
|  |  |  |  |
| Total | 317 (100) | 203 (100) | 520 |

P = 0.003

B

| **CT-scan availability** | **ICU type** | | **Total** |
| --- | --- | --- | --- |
|  | Public | Private |  |
|  | n (%) |  |  |
| <2 h | 200 (49) | 199 (68) | 399 (57) |
| 2-6 h | 123 (30) | 72 (24) | 195 (28) |
| >6 h | 80 (20) | 22 (7) | 102 (15) |
| Unavailable | 5 (1) | 1 (0) | 6 (1) |
|  |  |  |  |
| Total | 408 (100) | 294 (100) | 702 |

P < 0.001
